# Molecular Form Differences Between Prostate-Specific Antigen (PSA) Standards Create Quantitative Discordances in PSA ELISA Measurements

**DOI:** 10.1038/srep22050

**Published:** 2016-02-25

**Authors:** Erica L. McJimpsey

**Affiliations:** 1 National Institute of Standards and Technology, Material Measurement Laboratory, Gaithersburg, Maryland, 20878, USA.; 2 Department of Chemistry, Western Illinois University, Macomb, Illinois, 61455, USA.

## Abstract

The prostate-specific antigen (PSA) assays currently employed for the detection of prostate cancer (PCa) lack the specificity needed to differentiate PCa from benign prostatic hyperplasia and have high false positive rates. The PSA calibrants used to create calibration curves in these assays are typically purified from seminal plasma and contain many molecular forms (intact PSA and cleaved subforms). The purpose of this study was to determine if the composition of the PSA molecular forms found in these PSA standards contribute to the lack of PSA test reliability. To this end, seminal plasma purified PSA standards from different commercial sources were investigated by western blot (WB) and in multiple research grade PSA ELISAs. The WB results revealed that all of the PSA standards contained different mass concentrations of intact and cleaved molecular forms. Increased mass concentrations of intact PSA yielded higher immunoassay absorbance values, even between lots from the same manufacturer. Standardization of seminal plasma derived PSA calibrant molecular form mass concentrations and purification methods will assist in closing the gaps in PCa testing measurements that require the use of PSA values, such as the % free PSA and Prostate Health Index by increasing the accuracy of the calibration curves.

For almost two decades, immunoassays that measure the serum level of the biomarker, PSA, have been used for the early detection and therapeutic monitoring of prostate cancer (PCa)^1^. PSA, independently discovered by several researchers^2^ and in 1979 purified from prostatic tissue^3^, is an N-linked glycoprotein^4^ comprised of a 237 amino acid residue (28,400 Daltons (Da)). The predominant immunoreactive forms of PSA, also known as isoforms, that have been identified in serum include free (uncomplexed) PSA (fPSA) and PSA complexed to alpha 1-antichymotrypsin (PSA-ACT). It has been demonstrated that in men who have PCa, PSA-ACT is elevated^5^. Contrarily, benign prostatic hyperplasia (BPH), a benign enlargement of the prostate, is associated with higher non-intact, free PSA serum levels. High false positive rates^6^, problems with antibody detection of free and complexed PSA in equal molar ratios (equimolarity)^7^, as well as, measured PSA value discordances between immunoassay manufacturers^8^ have been the subject of controversy leading to great debate as to whether use of the test is of benefit. Identifying the source of inaccuracy in PSA measurements would greatly assist with increasing the specificity of PCa testing. The calibration curve of a PSA immunoassay plays a critical role in the accurate measurement of an unknown mass concentration of serum PSA. The calibration standards used in clinical and research grade PSA immunoassays are either free PSA, a ratio of free and complexed PSA, or PSA of a recombinant form. Non-recombinant PSA protein standards are purified from seminal plasma. In addition to precursor forms of PSA, seminal plasma contains free PSA that is composed of enzymatically active, intact PSA and enzymatically inactive, nicked and clipped (internally cleaved) molecular subforms^9^. These free PSA subforms are typically internally cleaved between residues 85–86, 145–146, and 182–183^10^,^11^. Since seminal plasma derived PSA standards are purified from pooled (i.e., multiple donors) specimens^12^, PSA immunoassay calibrants can potentially contain differences in the concentrations of intact PSA, as well as, PSA molecular subforms. One possible source for the lack of reliability in PSA immunoassays may be due, in part, to molecular differences in seminal plasma purified PSA calibrants. The objective of this study was to determine if differences in the composition of intact PSA and cleaved PSA subforms create discordances in PSA ELISA mass concentration measurements. In this study, PSA and PSA-ACT standards of a known mass concentration from different commercial sources were used as control standards (CS) and comparatively examined in several research grade total PSA (t-PSA) ELISAs. These immunoassays served as model clinical systems. Two of the assay calibrants, one of which was a recombinant PSA, were also comparatively investigated.

## Methods

### Disclaimer

Certain commercial equipment, instruments, and materials are identified in this paper in order to specify the experimental procedures adequately. Such identification is not intended to imply recommendation or endorsement by the NIST.

### Ethics Statement

“The NIST Institutional Review Board (IRB) was established by the Director of NIST to review the ethical acceptability of all research conducted by NIST where human subjects are used or involved.” This study did not involve human subjects and was therefore not reviewed by the NIST IRB. The experimental protocols were approved by line management in the Analytical Chemistry Division of the Material Measurement Laboratory at the NIST. The prostate cancer serum samples used for the study were collected with informed consent and in accordance with the BioServe Biotechnologies, Limited, IRB approved protocol.

#### Total PSA ELISA Tests

Three t-PSA ELISA kits (Calbiotech, #PS067T; R&D, #DKK300; Biocheck, #BC1029) were employed to evaluate PSA and PSA-ACT purified standards from different commercial sources. PSA calibrants from both the Calbiotech and R&D ELISAs were also examined. The Biocheck immunoassay was equimolar. The Calbiotech and R&D assays were non-equimolar. Immunoassay testing and kit storage was performed according to each manufacturers recommendation. Standards and calibrants were tested in triplicate. A Safire 2, Tecan Microplate Reader was utilized to collect raw absorbance values for all for the PSA standards and tested calibrants at a wavelength of 450 nm. Data analysis were performed in Excel 2011.

#### Commercial PSA and PSA-ACT CS

**Figure 1:**
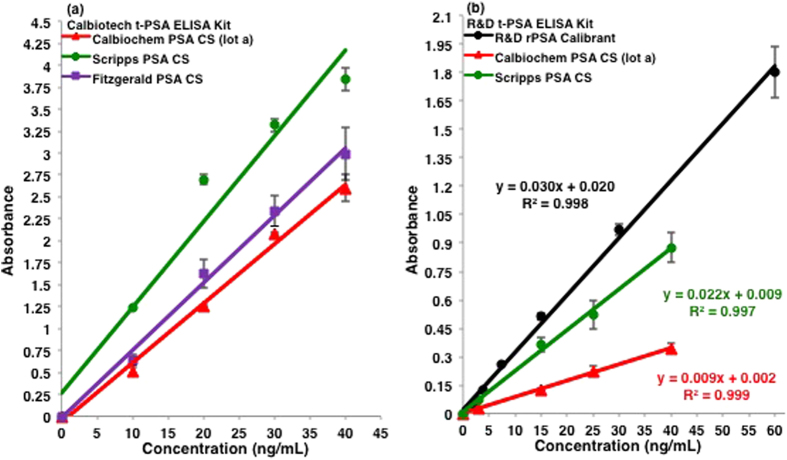
Total PSA ELISA comparisons of free PSA CS. Average absorbance values are plotted against the commercially reported mass concentrations for three free PSA CS (Calbiochem, Scripps, and Fitzgerald) in the (**a**) Calbiotech and (**b**) R&D t-PSA immunoassays. One standard deviation is shown for each plotted test point. In both immunoassays, all of the PSA standards had different absorbance values.

**Figure 2:**
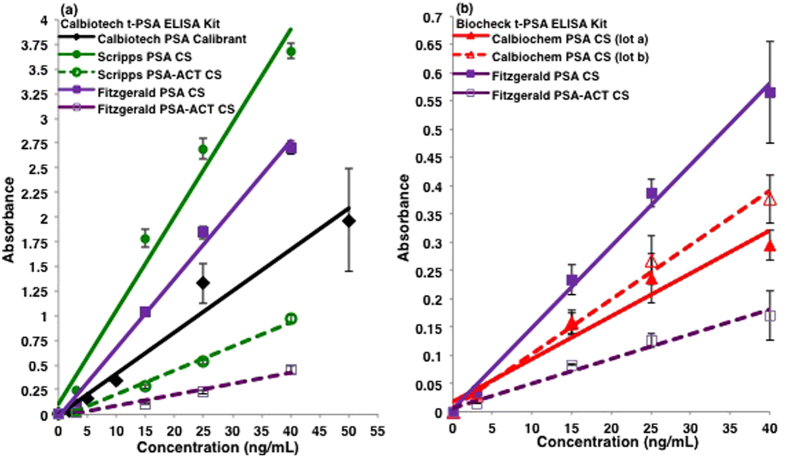
Calbiotech and Biocheck t-PSA ELISAs of free and complexed PSA CS. Lower absorbance values were observed with the PSA-ACT CS in comparison to the PSA CS in both the (**a**) Calbiotech and (**b**) Biocheck t-PSA immunoassays. One standard deviation is shown for each plotted test point. In both immunoassays, the measured absorbance values for all of the PSA and PSA-ACT standards were different.

**Figure 3:**
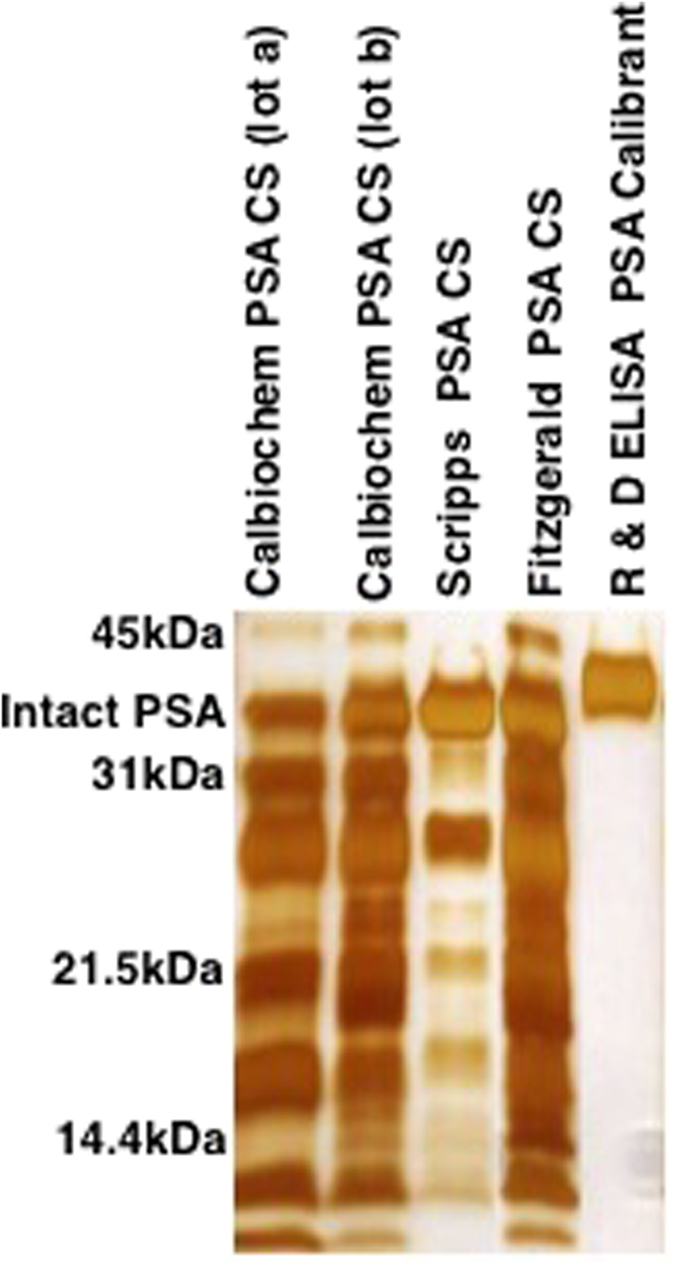
SDS-PAGE of free PSA standards. Differences in the relative concentrations of PSA isoforms and subforms were observed by SDS-PAGE for the free PSA CS from Calbiochem (2 different lots), Scripps, and Fitzgerald, as well as, the R&D ELISA rPSA calibrant.

Four free PSA (Calbiochem, #539832 (lots D10010682 and D00116361); Scripps, #P0725; and Fitzgerald, #30R-AP019) and two PSA-ACT (Scripps, #P0625 and Fitzgerald, #30-AP13) CS were studied. The standards tested in the Calbiotech t-PSA ELISA kit (Calbiochem PSA (lot a), Scripps PSA, Scripps PSA-ACT, Fitzgerald PSA, and Fitzgerald PSA-ACT) were serially diluted with an in-house prepared 50 mM ammonium bicarbonate (Sigma, #09830) solution containing 0.067% bovine serum albumin (Thermo Scientific, #23210) and a protease inhibitor cocktail (Thermo, #78430) diluted to manufacturer recommendations. The standards tested in the R&D assay (Calbiochem PSA (lot a) and Scripps PSA) and Biocheck assay (Calbiochem PSA (lots a and b), Fitzgerald PSA, and Fitzgerald PSA-ACT) were serially diluted with the kit manufacturer’s diluents. Final dilution concentrations for the CS were either ((10, 20, 30, and 40) ng/mL) (Fig. 1a) or ((3, 15, 25, and 40) ng/mL) (Figs 1b and 2a,b).

#### Prostate Cancer Samples

Two serum samples were purchased from the biorepository, BioServe Biotechnologies, Limited. Blood collection was performed with informed consent and in accordance with the Institutional Review Board approved protocol. Samples from PCa donors with pathologic T stages of 2c (sample 1, #112417) and 3b (sample 2, #115218) were tested in duplicate using the R&D immunoassay.

#### Gel Electrophoretic Methods

##### One-Dimensional PSA Gel

Two different lots of the Calbiochem PSA (D10010682 (lot a) and D00116361 (lot b)) CS, as well as, the Scripps and Fitzgerald PSA CS were studied by sodium dodecyl sulfate-polyacrylamide gel electrophoresis (SDS-PAGE). The R&D assay PSA calibrant (R&D, #1344-SE), prepared according to manufacturer recommendations, was also examined (Fig. 3). SDS-PAGE was performed according to Laemmli^13^ with the below modifications. Samples (3 μg) were diluted in a 1 to 1 ratio with buffer (Bio-Rad Laboratories, #161–0737) under reducing conditions with 5% β-mercaptoethanol. PSA samples and 10 μL of protein molecular mass standards (Bio-Rad, #161–0137) were boiled for 5 minutes. After heating, samples were loaded on a 1.0 mm thick, 12.5%T/3.3%C polyacrylamide gel. Gels were run in a Mini Protean Tetra Electrophoresis Cell (Bio-Rad, #165–0827) and a voltage of 80 V for 15 min followed by 80 min at 120 V was applied. Proteins were silver stained and destained (Thermo, #24612) according to manufacturer recommendations.

##### Western Blots of PSA and PSA-ACT

**Figure 4:**
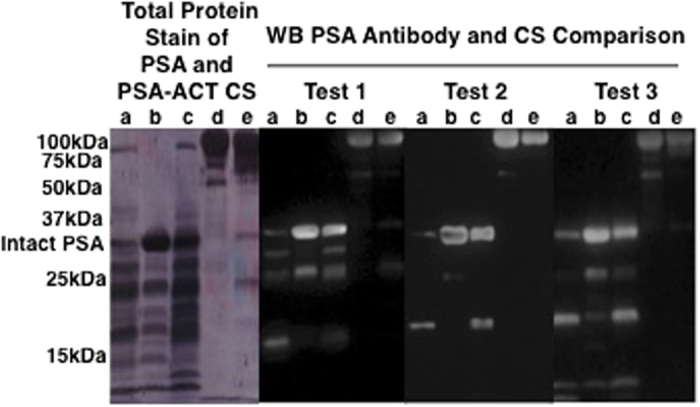
WB of free and complexed PSA CS. A total protein membrane stain of the PSA and PSA-ACT CS after WB test 1 is shown. WB studies using three different antibodies against t-PSA (Test 1, 2, and 3) for PSA CS from a) Calbiochem (lot a), b) Scripps, and c) Fitzgerald, as well as, PSA-ACT CS from d) Scripps and e) Fitzgerald are shown.

SDS-PAGE was performed on molecular mass standards (Bio-Rad, #161–0375), PSA CS (Calbiochem (lot a), Scripps, and Fitzgerald), and PSA-ACT CS (Scripps and Fitzgerald) as described above. Gel electrophoresis was run at a voltage of 80 V for 15 min followed by 70 min at 120 V. Gel protein bands were transferred onto polyvinylidene fluoride (PVDF) membranes (Bio-Rad, #162–0177) using a tank blotting system (Bio-Rad, #165–0827) in a 20% MeOH/buffer solution at a constant voltage of 60 for 2 hours. A western blot (WB) detection system (GE Healthcare, RPN2135) was used according to manufacturer recommendations. PVDF membranes were blocked with a 2% blocking agent (Bio-Rad, #170–6404) for 45 min. WB primary antibodies were diluted at a ratio of 1:5000 (Abcam, ab28563), 1:900 (Santa Cruz (SC) Biotechnology Incorporated, CHYH1, sc-69663), and 1:1600 (SC, C-19, sc-7638) for antibody tests 1, 2, and 3, respectively (Fig. 4). The corresponding secondary antibodies were diluted 1:100000 (SC, sc-2004), 1:2900 (SC, sc-2005), and 1:18500 (SC, sc-2768) for antibody tests 1, 2, and 3, respectively. WB PVDF membrane imaging was performed on an Alpha Innotech Fluorchem SP Imager. A total protein stain (Bio-Rad, #170–6527) was applied overnight to membranes from the WB antibody tests to confirm complete transfer of protein.

## Results

As shown in Fig. 1, the CS have different absorbance signals when tested in the Calbiotech and R&D immunoassays. The analytical response in absorbance for each of the three CS analyzed in the Calbiotech assay varied; the strongest absorbance signal was observed with the Scripps PSA while the Calbiochem PSA (lot a) exhibited the weakest. As was the case with the Calbiotech assay (Fig. 1a), the Scripps PSA had higher measured absorbance values relative to the Calbiochem PSA in the R&D assay (Fig. 1b); the recombinant PSA (rPSA) calibrant had the highest absorbance signals. Two PCa serum samples were also tested in the R&D assay. The absorbance values for the t-PSA blood levels are shown in Table 1. The t-PSA concentrations were computed using the linear regression equations of the Calbiochem and Scripps PSA standards, as well as, the rPSA immunoassay calibrant; the concentrations ranged between 4.0 ng/mL and 17.4 ng/mL for PCa serum sample 1 and 38.9 ng/mL and 133.5 ng/mL for sample 2. Complexed PSA was also studied in the Calbiotech (Fig. 2a) and Biocheck (Fig. 2b) assays. In the Calbiotech assay, both the Scripps and Fitzgerald PSA standards absorbed more strongly than the Scripps and Fitzgerald PSA-ACT standards (Fig. 2a). A comparison of the Calbiotech and Biocheck immunoassays yielded similar results for the Fitzgerald PSA and PSA-ACT standards. In the Biocheck ELISA (Fig. 2b), an equimolar assay, the absorbance signal of the Fitzgerald PSA-ACT standard was attenuated in comparison to the Fitzgerald PSA CS. Lot to lot variations in the Calbiochem PSA were also examined in the Biocheck assay (Fig. 2b). The Calbiochem PSA CS (lot b) had slightly higher absorbance values than the Calbiochem PSA CS (lot a).

To determine the source of the absorbance variances between the PSA standards, a 1D SDS-PAGE was performed on both of the Calbiochem PSA CS lots, as well as, the Scripps and Fitzgerald standards (Fig. 3). The R&D r-PSA calibrant was also examined. The intact PSA bands, observed at approximately 34 kDa, were higher than the molecular mass of free PSA; glycosylated proteins are known to migrate differently than non-glycosylated proteins on SDS-PAGE. The relative subform abundances of the two Calbiochem PSA CS lots varied and more intact PSA was observed in lot b than lot a. Similarly, the Scripps and Fitzgerald PSA standards had varying compositions of PSA molecular forms. The R&D r-PSA calibrant contained only the intact molecular form of PSA. PSA molecular form compositions were also examined by WB (Fig. 4). WB tests were performed on the three PSA and two PSA-ACT CS studied by ELISA using three different antibodies against both PSA and PSA-ACT (WB tests 1, 2, and 3). One representative total protein membrane stain, confirming the complete transfer of PSA standard, is shown for WB test 1 (Fig. 4). Antibody dependent differences in the presence and/or absence of PSA subforms were observed for each of the standards. Intact PSA was observed at approximately 34 kDa (Fig. 4a–c). Through the collective use of three PSA antibodies, up to 8 unique bands ranging in molecular mass between approximately 10 and 31 kDa were confirmed as subforms for each free PSA standard. Intact PSA-ACT bands were observed at approximately 100 kDa (Fig. 4d,e). PSA, as well as, PSA-ACT subforms were also present in the PSA-ACT standards (Fig. 4d,e). The relative amount of intact PSA present was determined through visual inspection and based on WB protein band size (Fig. 4). Both the intact PSA and cleaved subform concentrations varied by commercial source. The relative concentrations of intact free PSA observed between manufacturers ([Calbiochem PSA (lot a)]<[Fitzgerald PSA]<[Scripps PSA]) were consistent for each of the three PSA antibodies studied (Fig. 4). Similarly, the relative concentration of intact Scripps PSA-ACT was determined to be higher than that of the Fitzgerald PSA-ACT CS.

## Discussion

The PSA standard absorbance trends observed between assays were determined to be correlated with the concentration of intact PSA and PSA-ACT present in the standards, even between lots, in the case of the Calbiochem PSA standards (Fig. 2a). In the Calbiotech assay (Fig. 1a), the intensity of the free PSA standard absorbance signals from weakest to strongest was Calbiochem PSA (lot a)<Fitzgerald PSA<Scripps PSA. The WB studies (Fig. 4) revealed that the relative concentrations from lowest to highest of intact PSA for the PSA standards was [Calbiochem PSA (lot a)]<[Fitzgerald PSA]<[Scripps PSA]). When measured in the R&D assay (Fig. 1b), the PSA absorbance trends were Calbiochem PSA (lot a)< Scripps PSA<R&D rPSA. The R&D r-PSA calibrant was determined by SDS-PAGE to be composed solely of intact PSA and did not contain any internally cleaved subforms whereas the Scripps and Calbiochem PSA CS were composed of many (Fig. 3). In the Biocheck assay (Fig. 2b), the Fitzgerald PSA standard had higher absorbance signals in relation to the Calbiochem PSA standards for both lots a and b. The Calbiochem PSA lot b standard, however, had higher immunoassay absorbance values (Fig. 2b) and a higher relative concentration of intact PSA in comparison to lot a (Fig. 3). Lot to lot differences in the concentration of intact PSA between PSA standards from the same company can therefore create immunoassay absorbance differences. The Fitzgerald and Scripps PSA and PSA-ACT standards had similar absorbance and intact molecular form concentration trends. In the Calbiotech assay (Fig. 2a), the Scripps PSA-ACT standard had a higher absorbance than the Fitzgerald PSA-ACT CS. By WB, the concentration of the intact Scripps PSA-ACT was observed to be higher than that of the Fitzgerald PSA-ACT standard. In short, increased concentrations of intact PSA and PSA-ACT in the standards resulted in increases in immunoassay absorbance values. Lower concentrations of intact PSA and PSA-ACT in standards resulted in lower immunoassay absorbance values. These findings suggest that the PSA immunoassay antibodies are preferentially binding to the intact PSA and intact PSA-ACT molecular forms.

Although the WHO First International Standards for PSA (90% PSA-ACT: 10% free PSA (also known as 90:10) and free PSA) were established as calibrants to standardize PSA diagnostic immunoassays that were nonequimolar in response to PSA and PSA-ACT^14^, interassay disparities in free^15^ and total PSA measurements have continued^16^,^17^,^18^. In the Biocheck t-PSA ELISA results presented herein, the absorbance signal of the Fitzgerald PSA-ACT standard was attenuated in comparison to the Fitzgerald PSA standard, despite assay equimolarity. In addition to immunoassay nonequimolarity, some possible other causes for these absorbance differences include dissociation of PSA-ACT^19^ and/or inaccurate assignment of PSA-ACT mass concentration values^20^. A large majority of the research grade and diagnostic PSA immunoassays are likely not equimolar in response to the intact molecular forms and cleaved subforms of PSA and PSA-ACT.

The WHO PSA standards have been reported to yield 20–25% lower t-PSA and fPSA test results when calibrated against the Hybritech standards in the Access Immunoassay System^21^. In a study spearheaded by the UK National External Quality Assessment Service, the equimolar response of 11 diagnostic PSA immunoassays in use at 197 England laboratories was measured using different ratios of the WHO free and complexed PSA standards. None of the 11 assays were determined to be equimolar^7^. It is possible that in this study, the different ratios of PSA and PSA-ACT, though equal in PSA concentration, contained varying amounts of intact PSA and PSA-ACT, thereby resulting in equimolar disparities. These same diagnostic immunoassays, including but not limited to, Abbott Architect, Bayer Immuno 1, Beckman Hybritech, Perkin Elmer DELFIA, Roche E170, and Ortho Clinical Diagnostics are FDA approved and in use within the United States.

The observed molecular form composition differences between seminal plasma derived PSA standards, specifically the concentration of intact PSA, are likely due to the fact that PSA standards are purified from pooled specimens which will vary in accordance with the individual donor’s intact PSA and cleaved subform makeup. The use of various purification processes of PSA by different manufacturers will further alter the composition of the PSA standard molecular forms present. Though not tested in this study, lot to lot differences in the concentration of intact PSA, would be possible in the two WHO PSA standards (90% PSA-ACT: 10% free PSA and free PSA), as each one contains free PSA purified from seminal plasma.

The PCa serum sample PSA value findings from this study (Table 1) demonstrate that molecular form differences between PSA standards can create intraassay discordances in t-PSA measurements that could ultimately result in unreliable PSA serum level reporting from clinical laboratories. For PCa serum sample 1, the computed t-PSA concentrations ranged between 4.0 ng/mL and 17.4 ng/mL, depending on the calibration curve used. Since the free PSA PCa serum levels were not tested, the corresponding free-to-total PSA ratios (free PSA / free PSA + PSA-ACT), also known as % fPSA, are not available for review. Any deviations, however, from accuracy of the t-PSA (free PSA + PSA-ACT) serum concentration would also result in inaccurate % fPSA (free PSA/free PSA + PSA-ACT) reporting. It is worth reminding the reader that % fPSA, the measurement used to differentiate BPH from PCa^22^, is a calculation of the PSA concentrations from two different PSA immunoassays that rely on two distinct calibration curves and PSA calibrants to determine the unknown free and complexed PSA serum levels. The cumulative effects of the experimental differences found between the free and t-PSA immunoassays, due to variations in standards, challenges the utility of % fPSA numerical ratio values.

Use of any nonequimolar PSA immunoassay that contains a seminal plasma derived PSA calibrant could result in skewed PSA serum level reporting leading to either overdiagnosis or underdiagnosis of PCa. Over the past 15 years, many derivatives of the PSA test have been developed to increase the specificity of PCa detection, including but not limited to, % fPSA, PSA velocity (PSAV), and the prostate health index (PHI). The PSAV, a measure of the rate of change in PSA levels, has been shown to only minimally, in comparison to % fPSA, increase the predictive accuracy of early PCa detection (AUC of 0.626 vs 0.609)^23^. The PHI, a newly FDA approved test for the early detection of PCa in the 2.0 to 10.0 ng/mL PSA range^24^, has shown more promise. This test utilizes a score based on the mathematical formula, ([−2]proPSA/free PSA) × √PSA), which combines results from three individual PSA measurements (t-PSA, % fPSA, and a truncated precursor form of fPSA containing a leader sequence of 2 amino acids known as [−2]proPSA). Although at a sensitivity of 90%, Jansen *et al.* determined the specificity of PHI to be 31%, in comparison to 11% and 10% for % fPSA and t-PSA, respectively, a gap in the reduction of false positives still exists^25^. The overall improvement in screening of PCa using the PHI makes sense; the [−2]proPSA measurement is performed through use of a recombinant [−2]proPSA calibrant and an anti [−2]proPSA antibody that exhibits low crossreactivity to free PSA (<0.2%) and other truncated precursor forms of PSA (<2%)^26^. This high specificity of the anti [−2]proPSA antibody to [−2]proPSA removes potential downstream problems of preferential binding to the antibody from other serum PSA molecular forms. Use of an accurate [−2]proPSA calibration curve, as well as, the accurate measurement of serum [−2]proPSA would therefore result in increased specificity of the PHI test in the discrimination of PCa. As it stands currently, PCa screenings that require longitudinal monitoring of PSA, such as PSAV and PSA doubling time are only of diagnostic benefit if with each repeated measurement, the clinical laboratory, PSA assay, and molecular form concentrations of the PSA and PSA-ACT calibrant remain exactly the same.

Until PSA immunoassays are equimolar in response to intact PSA and PSA-ACT, as well as, the precursor forms and cleaved molecular subforms of PSA and PSA-ACT, standardization of molecular form mass concentrations to known and absolute values is needed in seminal plasma derived PSA calibrants to help reduce PSA level reporting errors. Upon determination of the molecular form ratios and mass concentrations present in serum fPSA, as well as, serum fPSA complexed to ACT during early stage PCa, better standardization of PSA immunoassay calibrants could be achieved. Preparation of a fPSA calibrant would require a multistep process and include seminal plasma purification, isolation, and quantification of the previously determined fPSA molecular forms present in PCa followed by recombination of the molecular forms in defined mass concentrations. The same general protocol for preparation of a PSA-ACT calibrant could be followed with the exception that complexation of the fPSA to ACT would be needed prior to combining the PSA molecular forms. For the t-PSA immunoassay, use of a calibrant containing the already adopted isoform ratio of 90% PSA-ACT and 10% fPSA with the previously identified molecular form ratios and mass concentrations for each isoform would best be employed.

## Conclusion

The intact PSA and intact PSA-ACT molecular forms of the PSA standards examined in this study were observed to preferentially bind in several t-PSA immunoassays, regardless of assay equimolarity. Diagnostic use of PSA immunoassays that exhibit a nonequimolar response to any of the various molecular forms of PSA and PSA-ACT could result in unreliable PSA serum level reporting, if the immunoassay calibrants are not standardized to known and absolute molecular form ratios and mass concentrations. Molecular form ratio and mass concentration standardization of PSA immunoassay calibrants is possible and would assist in increasing the specificity of PCa testing. The same would be true for BPH. To achieve better uniformity between laboratories, standardization of a seminal plasma PSA purification protocol would also be of benefit. A standardized PSA test method is also needed.

## Additional Information

**How to cite this article**: McJimpsey, E. L. Molecular Form Differences Between Prostate-Specific Antigen (PSA) Standards Create Quantitative Discordances in PSA ELISA Measurements. *Sci. Rep.* **6**, 22050; doi: 10.1038/srep22050 (2016).

## Figures and Tables

**Table 1:** PCa serum t-PSA measurement comparison.

PCa Serum Samples	Pathologic Tumor Stage of PCa Serum Donor	Measured Absorbance of t-PSA	Coefficient of Variation	t-PSA (ng/mL) Measurement Comparison Using Linear Regression Equations from R&D Immunoassay PSA Calibrant and CS		
				R&D rPSA Calibrant	Calbiochem PSA CS	Scripps PSA CS
1	2c	0.80	9.6	4.0	17.4	6.5
2	3b	0.60	1.7	38.9	133.5	54.0

The t-PSA serum levels of two PCa samples examined in the R&D ELISA were computed using the linear regression equations from the R&D ELISA rPSA calibrant and R&D tested PSA CS.
